# Computed Tomography Imaging in Simulated Ongoing Cardiopulmonary Resuscitation: No Need to Switch Off the Chest Compression Device during Image Acquisition

**DOI:** 10.3390/diagnostics11061122

**Published:** 2021-06-19

**Authors:** Jessica Graef, Bernd A. Leidel, Keno K. Bressem, Janis L. Vahldiek, Bernd Hamm, Stefan M. Niehues

**Affiliations:** 1 Department of Radiology, Campus Benjamin Franklin, Charité–Universitätsmedizin Berlin, 12203 Berlin, Germany; keno-kyrill.bressem@charite.de (K.K.B.); janis.vahldiek@charite.de (J.L.V.); bernd.hamm@charite.de (B.H.); 2 Department of Emergency Medicine, Campus Benjamin Franklin, Charité–Universitätsmedizin Berlin, 12203 Berlin, Germany; bernd.a.leidel@charite.de; 3 Berlin Institute of Health at Charité–Universitätsmedizin Berlin, 10117 Berlin, Germany

**Keywords:** computed tomography, cardiopulmonary resuscitation, image quality, emergency medicine, automated chest compression

## Abstract

Computed tomography (CT) represents the current standard for imaging of patients with acute life-threatening diseases. As some patients present with circulatory arrest, they require cardiopulmonary resuscitation. Automated chest compression devices are used to continue resuscitation during CT examinations, but tend to cause motion artifacts degrading diagnostic evaluation of the chest. The aim was to investigate and evaluate a CT protocol for motion-free imaging of thoracic structures during ongoing mechanical resuscitation. The standard CT trauma protocol and a CT protocol with ECG triggering using a simulated ECG were applied in an experimental setup to examine a compressible thorax phantom during resuscitation with two different compression devices. Twenty-eight phantom examinations were performed, 14 with AutoPulse^®^ and 14 with corpuls cpr^®^. With each device, seven CT examinations were carried out with ECG triggering and seven without. Image quality improved significantly applying the ECG-triggered protocol (*p* < 0.001), which allowed almost artifact-free chest evaluation. With the investigated protocol, radiation exposure was 5.09% higher (15.51 mSv vs. 14.76 mSv), and average reconstruction time of CT scans increased from 45 to 76 s. Image acquisition using the proposed CT protocol prevents thoracic motion artifacts and facilitates diagnosis of acute life-threatening conditions during continuous automated chest compression.

## 1. Introduction

Every year, approximately 148,000 patients in Europe suffer out-of-hospital cardiac arrest, and more than 67% of them depend on life support, including cardiopulmonary resuscitation (CPR) and ventilation [1]. Automated chest compression (ACC) devices have been increasingly used in emergency settings over the last 15 years. Two common mechanical CPR devices are the corpuls cpr^®^ and AutoPulse^®^ devices. Even if the application of such devices is not considered superior to manual CPR, it is useful in circumstances in which constant high-quality manual CPR cannot be guaranteed, e.g., during patient transport, diagnostic procedures, or to support medical staff during prolonged CPR [2,3,4,5,6,7]. In the prehospital phase, the underlying cause of cardiac arrest is usually unknown and further diagnostics are required.

Radiological imaging plays a crucial role in detecting life-threatening conditions. The most common causes of sudden circulatory arrest are pulmonary embolism, acute aortic syndrome, hemorrhages, and cardiac events [8,9]. Each of these conditions is detected effectively by contrast-enhanced computed tomography (CT), which can thus improve patient outcome and survival [10,11]. In patients depending on CPR; however, diagnostic workup remains difficult. Therefore, it is common practice to interrupt CPR to obtain artifact-free CT images and protect medical staff from radiation exposure [12,13,14]. On the other hand, interruption of CPR may not only worsen patient outcome but also degrade the quality of the acquired images [5,15,16,17,18,19,20]. During circulatory arrest, the contrast agent is not distributed sufficiently, reducing image quality and complicating diagnostic assessment of vascular structures.

To overcome such limitations and minimize time without CPR, mechanical CPR devices can be left in the gantry during a CT examination [12,13,21,22,23]. However, as the devices contain several metal components, they can lead to beam hardening artifacts affecting diagnostic evaluation of major structures of interest in the mediastinum (pulmonary arteries, coronary arteries and ascending aorta). Furthermore, repeated chest compressions generate additional extensive motion artifacts, further reducing image quality and possibly leading to non-diagnostic images.

Therefore, the aim of this study was to evaluate a CT protocol allowing continuous CPR during CT imaging while reducing motion artifacts and as a result improving diagnostic assessment of central chest structures.

## 2. Materials and Methods

### 2.1. Body Phantom

The phantom used in the experiments consisted of a compressible chest model for resuscitation training (Laerdal Medical GmbH, Puchheim, Germany) and a heart equivalent filled with diluted contrast agent (30 mL iomeprol (400 mg I/mL, Imeron 400, Bracco Imaging, Milan, Italy) and 150 mL of 0.9% sodium-chloride solution). The heart model featured a visible cardiac wall and an air bubble to simulate a small abnormality such as thrombus or intraventricular air. All CT images were acquired with the same phantom. The ACC devices were placed for optimal operation as recommended by the manufacturers.

### 2.2. CT Examinations

Two different CT scanning protocols were used for comparing image quality, reconstruction time as well as radiation dose. All examinations were performed on a 320-slice multidetector CT scanner (Aquilion ONE Genesis, Canon Medical Systems, Otawara, Japan). Technical parameters were: tube voltage of 120 kV, modulated tube current (100–700, SD = 10), rotation time of 0.275 s, pitch factor of 0.814, and collimated slice thickness of 80 mm × 0.5 mm.

### 2.3. CT Protocols

**Figure 1:**
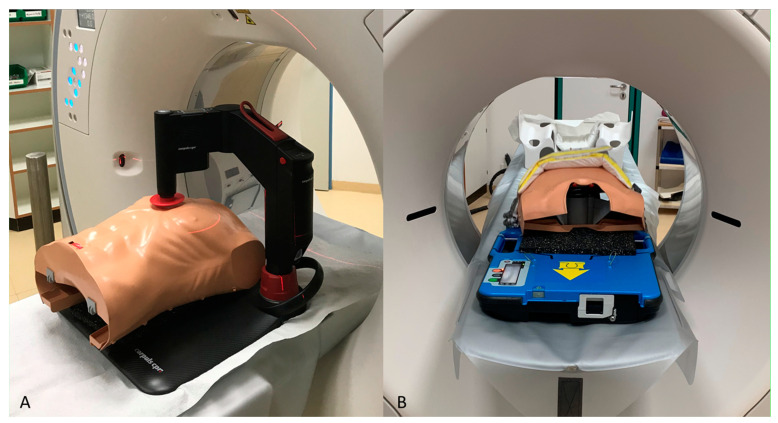
Setup of the phantom examinations using corpuls cpr (**A**) and AutoPulse (**B**).

Two different CT protocols were investigated in this study: Protocol A represented the hospital’s internal standard protocol for CT examinations of severely injured patients. For protocol B, an ECG signal of the same frequency as the ACC device was simulated. A retrospective virtual ECG-gated scan with automatic best-phase detection was then applied in order to achieve artifact-free CT images during ongoing mechanical resuscitation. For the CT examinations with both protocols, the ACC device was placed in the CT gantry together with the phantom. The images were acquired while automated chest compressions at the recommended frequency of each ACC device were performed (Figure 1).

Twenty-eight phantom examinations were performed, 14 during resuscitation with Autopulse and 14 with corpuls cpr. For each mechanical CPR device, seven CT examinations were carried out with the standard protocol and seven with ECG triggering. All results were acquired under identical conditions and pooled across the seven scans. The following CT-related data were collected for analysis: protocol type (triggered, not triggered), dose-length product (DLP) in mGy·cm, and reconstruction time.

All CT datasets were stored in the local Picture Archiving and Communication System (PACS) for subsequent analysis. For reading and generating dynamic clips and volume-rendered images, dedicated software was applied (Visage 7, Version 7.1, Visage Imaging, San Diego, CA, USA).

### 2.4. Automated Chest Compression Devices and Synchronization

Two frequently used automated chest compression devices were employed to investigate the quality of CT images acquired during resuscitation. The corpuls cpr (GS Elektromedizinische Geräte G. Stemple GmbH, Kaufering, Germany) used a stamp mechanism with a frequency of 100 compressions per minute as recommended in the 2015 version of the European Resuscitation Council Guidelines for Resuscitation [6]. The AutoPulse Resuscitation System (ZOLL Medical Deutschland GmbH, Cologne, Germany) compressed the chest circumferentially at a frequency of 80 compressions per minute using a chest belt. Each of the two devices was placed around the phantom following the manufacturers’ instructions and compressed the sternum (corpuls cpr) or the entire chest (AutoPulse).

The ECG was simulated by means of a cardiac trigger monitor (Cardiac Trigger Monitor 7800, Ivy Biomedical Systems, Inc., Branford, CT, USA). For both mechanical CPR devices, the trigger monitor simulated an ECG signal according to the frequency used by the device (100 compressions per minute for corpuls cpr and 80 compressions per minute for AutoPulse).

### 2.5. Image Quality

Analysis of image quality focused on the ability to delineate anatomic structures of the heart equivalent (cardiac wall and intraventricular pathology) and the chest wall of the phantom, matching the skin contours of humans in CT examinations. Image quality was evaluated in consensus by two readers with different levels of CT reading expertise. For subjective assessment, the readers used a four-point Likert scale to classify the severity of motion artifacts induced by chest compressions:

- 1 = no artifacts
- 2 = artifacts without impairment of image quality
- 3 = artifacts with moderate impairment of image quality
- 4 = artifacts with severe impairment of image quality

### 2.6. Radiation Dose

To identify possible differences in radiation exposure between the investigated CT protocols, the estimated radiation dose of each scan, the dose-length product (DLP) in mGy·cm, and the effective dose (E) in mSv were determined. The effective dose was calculated by applying the method and conversion coefficients described in the European Guidelines on Quality Criteria for Computed Tomography to the DLP. The formula was E = k × DLP, with a mean conversion coefficient (k) for the chest of 0.017 [24].

### 2.7. Statistical Analysis

Statistical analysis was performed using SPSS (SPSS^®^, v. 25.0; IBM Corp., Armonk, NY, USA). The Kolmogorov–Smirnov test was applied to test for normal distribution. Normally distributed variables were reported as mean ± standard deviation. Non-symmetrically distributed variables were presented as median and were compared by means of the Mann–Whitney U-test. Minimum and maximum were presented for all quantitative variables. Likert scales were compared using the Wilcoxon signed-rank test. A level of *p* < 0.05 was considered to indicate statistically significant differences.

## 3. Results

An overview of the two CT protocols used in the phantom experiments is presented in Table 1. Due to the experimental setup and the constant examination parameters, no variations of tube voltage, effective mA, and rotation time were observed in the respective protocols.

### 3.1. Image Quality

**Figure 2:**
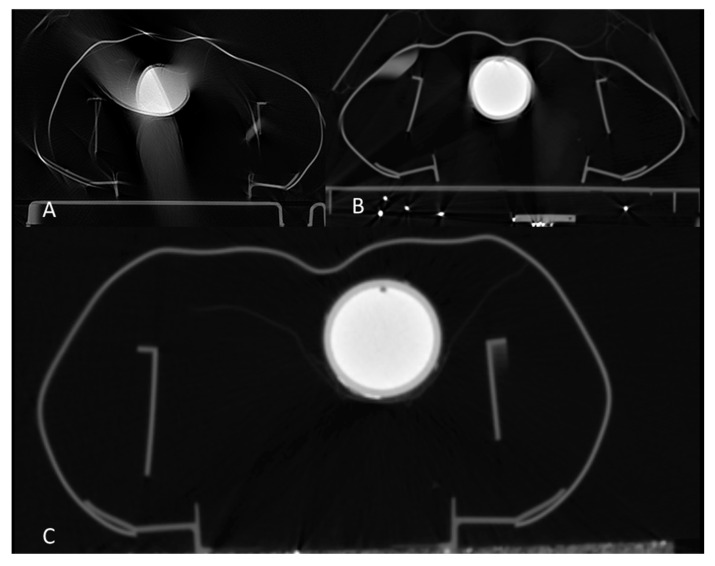
Examples of axial computed tomography images acquired with three different setups during ongoing cardiopulmonary resuscitation (CPR) with an automated chest compression device: standard trauma protocol during CPR with Autopulse (**A**) and ECG-triggered protocol during CPR using Autopulse (**B**) and corpuls cpr (**C**).

CT image quality differed significantly (*p* < 0.001) from the clinical CT trauma protocol (mean Likert scale of 3.93) and the ECG-triggered CT protocol (mean Likert scale of 1.14), while both protocols were carried out with the ACC devices in operation. Scores of 4 (indicating severe motion artifacts) were assigned to 92% of the CT datasets acquired with the trauma protocol, whereas scores of 1 (indicating excellent image quality without motion artifacts) were assigned to 85% of the CT datasets acquired using the ECG-triggered protocol (Figure 2).

Comparison of CT examinations acquired with both mechanical CPR devices revealed different image quality during resuscitation. The corpuls cpr setup (mean Likert score of 1.00) appeared to be superior to the AutoPulse setup (mean score of 1.29), although the limited number of evaluations did not allow further statistical analysis. The application of the triggered CT protocol in conjunction with the corpuls cpr device resulted in artifact-free images. In contrast, slight motion artifacts remained when CPR was performed with AutoPulse during CT scanning. Persistent artifacts were particularly visible in the area of the artificial cardiac wall despite the lower compression frequency of this device. However, the intraventricular pathology was visible in all CT images acquired during resuscitation with AutoPulse.

### 3.2. Radiation Dose

**Figure 3:**
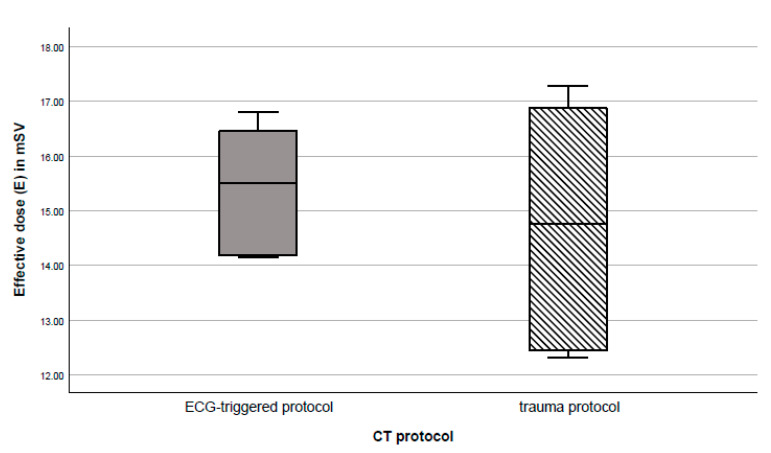
Comparison of medians and interquartile ranges of radiation dose (effective dose) with the two computed tomography protocols investigated. The difference is not statistically significant (*p* = 0.946).

The two investigated CT examination protocols differed in radiation dose, but the difference was not statistically significant (*p* = 0.946). The mean effective dose applied with the clinical trauma protocol during automated chest compression was 14.76 mSv (868.15 mGy·cm; minimum of 12.32 mSv, maximum of 17.28 mSv) versus 15.51 mSv (912.30 mGy·cm; minimum of 14.16 mSv, maximum of 16.81 mSv) with the protocol using ECG triggering. This corresponded with a 5.09% increase in radiation dose with the triggered protocol (Figure 3).

## 4. Discussion

The investigated protocol combining a simulated ECG during the CT scan with best-phase retrospective reconstruction significantly improved image quality during ongoing mechanical chest compressions at the cost of an insignificant increase in radiation dose. Another effect was an average 31-s increase in reconstruction time.

The findings showed that the ECG-triggered CT protocol presented here ensured acquisition of CT images of adequate diagnostic quality during ongoing mechanical CPR, allowing for fast and correct diagnosis in patients whose survival crucially depends on prompt initiation of adequate treatment.

To our knowledge, only a small number of case reports and one phantom study have so far been published on the use of ACC devices during CT examinations. However, in contrast to the experimental approach outlined above, all reported studies were conducted in setups with the ACC device placed in the CT gantry along with the patient but with discontinuation of mechanical CPR during acquisition of CT images. Accordingly, the presence of the device caused artifacts while image quality was still adequate for diagnosing underlying conditions [12,13,14]. However, to achieve this level of image quality, interruption of CPR was necessary, which was not the case with the presented protocol.

Wirth et al. obtained adequate image quality to identify the underlying conditions leading to circulatory arrest in all three patients while switching off the ACC device during acquisition of the diagnostic images [12]. Time without CPR was reduced by using automated chest compression while obtaining the scout view [12]. However, Wirth et al. also reported it was necessary to rerun the cranial CT of one patient due to the battery of the AutoPulse causing extensive artifacts and resulting in non-diagnostic image quality [12]. Using a chest phantom in our experimental setup, the occurrence of artifacts degrading image quality in other body regions could not be assessed. This aspect should be addressed in a future study using a whole-body model or a trunk-and-head phantom.

Schubert et al. also reported a case study of a patient who underwent a CT examination in circulatory arrest [13]. During acquisition of the scout view and distribution of the contrast agent, the ACC device was in operation but was then switched off for the CT examination proper [13]. Leidel et al. used manual compressions from behind or in front of the gantry for distributing the contrast agent [14]. The mechanical CPR device was also switched off during CT acquisition [14].

With the investigated protocol presented above, it is possible to trigger the CT with a simulated ECG during CPR. Using this approach it is technically not possible to directly connect the ACC device to the CT scanner and therefore, it is also not possible to select a certain reconstruction time within the R-to-R interval in the ECG. This was addressed by using the CT scanner’s automatic imaging raw-data-based motion recognition feature (“best phase”-detection) to identify an optimal phase with the least motion artifacts. Once activated within the protocol, this feature does not require any additional interaction.

CT scanners from other manufacturers might use a different approach, but in any case it would be possible to reconstruct the whole dataset, i.e., in a 5% to 10% interval. A 10% interval would increase the reconstruction time to 6.22 min, while the number of images would increase from 497 to 5467, both doubling in case the 5% steps are chosen. As time is of the essence in patients with circulatory arrest, this reconstruction method would be too time-consuming in most cases. It also requires additional interaction for choosing the optimal reconstruction set instead of evaluating images for underlying condition.

A possible approach to reduce radiation exposure would be an artificially generated frequency-adapted ECG, which is provided by the ACC device and allows prospective gating. Several studies have shown that the latter can reduce the radiation dose of cardiac CT scans by 67.3% to 84.9% compared to retrospective gating [25,26,27,28]. This setup would also reduce the time required to prepare the patient prior to the examination itself.

An additional approach would be the deployment of dedicated reconstruction techniques. Iterative reconstruction (IR) was introduced in 2009 and has made several improvements since its inception. This reconstruction technique provides a significant improvement in image quality due to considerable noise reduction [29,30,31]. It has also been investigated for CT examinations of critically ill patients requiring immediate CT diagnostics [32]. In addition, it revealed the potential for radiation dose reductions [32].

Therefore, the IR technique AIDR 3D (Canon Medical Systems, Otawara, Japan) was applied in all protocols mentioned in the study above. In general, the complex algorithms of this technique require more time for image reconstruction. Recent developments have introduced image reconstruction techniques based on deep-learning, allowing to reduce radiation exposure and improving image quality even more while maintaining significant noise reduction [33,34]. Regarding emergency diagnostics, time is one of the most relevant factors, and therefore, deep learning algorithms such as AiCE (Canon Medical Systems, Otawara, Japan) and TrueFidelity™ (GE Healthcare, Chicago, IL, USA) may have the potential to boost diagnostic quality and to reduce reconstruction time simultaneously.

A comparison of the two ACC devices tested in the presented study showed CT image quality of the Autopulse to be inferior to the image quality of the corpuls cpr. This observation is attributed to the different compression mechanisms (Autopulse using a chest belt versus corpuls cpr using a stamp). As the chest belt generates continuous chest motion, the time span of the chest being completely motionless is considerably shorter. However, the application of the best-phase algorithm resulted in images of sufficient diagnostic quality during resuscitation with both ACC devices.

## 5. Limitations

This study was performed as a proof-of-concept study of a functioning prototype for ECG-triggered CT acquisition during automated chest compression in patients with circulatory arrest. To further prove the feasibility of this approach, the results need to be confirmed in emergency settings in clinical practice. The requirement of a reliable routine in applying that kind of setup in an emergency case might represent a potential drawback. Therefore, further investigation under real-life circumstances with additional technical support is required in order to assess the developed method adequately. However, the significant gain in diagnostic accuracy might justify the additional effort. Furthermore, the setup presented here might not work with devices from other manufacturers, and therefore, the results of this study cannot be generalized at this point. The image quality was assessed in consent by two readers with different levels of reading expertise. Due to the small sample size the readers discussed the image quality in consent, but individual rankings of each reader as well as the interrater agreement cannot be provided.

## 6. Conclusions

The investigated protocol significantly reduced artifacts in CT images acquired during ongoing automated chest compression in the process of CPR, thus allowing for the evaluation of important thoracic structures and reliable diagnosis of major underlying causes of circulatory arrest.

## Figures and Tables

**Table 1:** Overview of computed tomography protocol characteristics.

	Protocol A Not ECG-Triggered	Protocol B ECG-Triggered
Reconstruction mode	half	best-phase
Examination mode	trauma protocol	retrospectively triggered protocol
Tube voltage [kV]	120	120
Effective mA	170	162
Rotation time	0.275	0.275
Total scan time [s] (min-max)	19 (18–20)	23 (22–24)
Reconstruction time [s] (min-max)	45 (43–48)	76 (73–81)

## Data Availability

The data presented in this study are available on request from the corresponding author.
